# Identifying different patterns of emotion dysregulation in adult ADHD

**DOI:** 10.1186/s40479-023-00235-y

**Published:** 2023-09-25

**Authors:** Emilie Martz, Luisa Weiner, Sébastien Weibel

**Affiliations:** 1 INSERM U1114, Strasbourg, France; 2 University of Strasbourg, Strasbourg, France; 3 Laboratoire de Psychologie Des Cognitions, University of Strasbourg, Strasbourg, France; 4 Psychiatry Department, University Hospital of Strasbourg, Strasbourg, France

**Keywords:** Adult ADHD, Emotion dysregulation, High-functioning ADHD, Factor analysis, Mono-dimensional clustering

## Abstract

**Background:**

Emotion dysregulation (ED) is a core intrinsic feature of adult presenting Attention Deficit Hyperactivity Disorder (ADHD). However, the clinical expressions of ED are diverse and several questionnaires have been used to measure ED in adults with ADHD. Thus, to date, the characteristics of ED in adult ADHD remain poorly defined. The objective of this study is to identify the different patterns of ED in adults with ADHD.

**Methods:**

A large sample of 460 newly diagnosed adults with ADHD were recruited. Patients completed a total of 20 self-reported questionnaires. Measures consisted in the several facets of ED, but also other clinical features of adult ADHD such as racing thoughts. A factor analysis with the principal component extraction method was performed to define the symptomatic clusters. A mono-dimensional clustering was then conducted to assess whether participants presented or not with each symptomatic cluster.

**Results:**

The factor analysis yielded a 5 factor-solution, including “*emotional instability*”, “*impulsivity*”, “*overactivation*”, “*inattention/disorganization*” and “*sleep problems*”. ED was part of two out of five clusters and concerned 67.52% of our sample. Among those patients, the combined ADHD presentation was the most prevalent. Emotional instability and impulsivity were significantly predicted by childhood maltreatment. The ED and the “*sleep problems*” factors contributed significantly to the patients’ functional impairment.

**Conclusions:**

ED in ADHD is characterized along emotional instability and emotional impulsivity, and significantly contributes to functional impairment. However, beyond impairing symptoms, adult ADHD may also be characterized by functional strengths such as creativity.

## Introduction

Attention Deficit Hyperactivity Disorder (ADHD) is a prevalent neurodevelopmental disorder, concerning 4% of the adult population [1–3]. ADHD involves difficulties in managing attention resources, motor restlessness and impulsive behaviors leading to major functional difficulties [4–6]. Three clinical pictures of ADHD have been classically described. The predominantly inattentive presentation concerns patients who meet difficulties in sustaining their attention, and present with distractibility and disorganization. The predominantly hyperactive presentation refers to patients who deal with motor restlessness, excessive talkativeness and difficulties in waiting their turn. The combined presentation corresponds to a combination of the forementioned symptoms [4]. However, when applied strictly, these criteria often lead to false negatives and underdiagnosis of ADHD in adults [7]. In fact, the clinical picture of ADHD in adults is not a perfect continuation of the ADHD symptomatology found in children. Indeed, in adults, attention deficits often increase, while motor hyperactivity tends to be internalized [7–11]. Thereby, many studies have suggested that adult ADHD should be considered beyond the classical triad of symptoms reported in children. For instance, based on patients’ reports, Asherson [12] described a specific clinical picture found in adults with ADHD, including inattention, hyperactivity, impulsivity, but also mental restlessness, sleep difficulties and, most importantly, emotion dysregulation (ED; [12, 13]). ED is characterized by the experience and expression of excessive emotions along with rapid and exaggerated swings in emotional states [14]. ED can be conceptualized as a dimensional construct as individuals differ in terms of both the number and the severity of ED symptoms (e.g. emotional lability, irritability, low frustration tolerance;14). From this perspective, given that ED is found in different psychiatric diagnoses, ED is usually considered as a transdiagnostic feature [15–17]. For instance, high levels of ED are found in Borderline Personality Disorder (BPD), but also in mood disorders, anxiety disorders and substance use disorders [17–21].

Since the first descriptions of adults with ADHD, emotional difficulties have been considered as a main symptom of the disorder in adults. For instance, Wender et al. [22] considered ED among the core symptoms of adult ADHD. This has been confirmed by several recent studies [23–26]. Indeed, ED is experienced by up to 70% of adults with ADHD [14, 27–29] and is highly associated with the core ADHD symptoms [30–34]. For instance, ED is particularly associated with hyperactivity [35], but also to additional symptoms typically found in adults with ADHD, i.e., racing thoughts and sleep difficulties [36]. Importantly, compared to the classic triad of symptoms, ED has been found to contribute significantly more to functional impairment in adults with ADHD [14, 24, 34, 37–39].

Regarding the etiology of ED in ADHD, it has been demonstrated that ED is not due to a comorbid disorder, but it is rather intrinsic to ADHD [34, 40–43]. However, little is known about the factors contributing to the development of ED in ADHD. To date, the genetic hypothesis is the most studied, considering that ADHD and ED are highly heritable [44–47]. Furthermore, family studies highlighted that the first-degree relatives of people presenting with both ADHD and ED are more likely to present with emotional difficulties than the first-degree relatives of individuals presenting with ADHD without ED [48–50]. However, some environmental factors might also be involved, such as childhood maltreatment. Indeed, compared to non-clinical controls, adults with ADHD report a greater number of traumatic events during childhood [51]. Conversely, maltreated children often present with ADHD [52, 53]. In a recent study led by Rüfenacht et al. [54], self-reported emotional neglect and abuse during childhood were found to contribute to emotional reactivity and poor emotion regulation strategies in adult ADHD.

Although ED is not considered to be disorder-specific, BPD is typically seen as the “*gold-standard*” presentation of ED. BPD is a highly impairing mental disorder characterized by a persistent pattern of unstable relationships associated with pronounced impulsive and self-harming behaviors, unstable identity and difficulties regulating emotions [4]. More specifically, ED in BPD is defined by affective instability, uncontrolled anger, and impulsive self-harming behaviors [55–58]. This clinical description of ED in BPD has been confirmed by studies using experience sampling assessments of emotional symptoms. In these studies, adults presenting with BPD have been found to experience more intense negative emotions, but also a greater instability of both positive and negative emotions compared to healthy peers [59–61]. Moreover, from a dynamic standpoint, adults with BPD experience emotional over-reactivity combined with a slower return to a neutral emotional state [62]. In adults with ADHD, a similar expression of ED (i.e. increased intensity and instability of negative emotions) has been reported in a number of studies [14, 41, 57, 63–66]. In particular, adults with ADHD present high levels of irritability, temper outbursts [41, 67], emotional lability (i.e. shifts from a neutral state to depressed mood or mild excitement; [12, 33, 34, 57, 67, 68]) and emotional over-reactivity (vulnerability to stressful situations, rapidly overwhelmed; [12, 33, 68]). However, compared to adults with ADHD, people with BPD experience reduced intensity of happiness [57].

Although the presence of ED in adults with ADHD is now established, the clinical characteristics of ED in ADHD remain poorly defined. Indeed, according to the instruments used in different studies, ED in ADHD has been characterized as “irritability” [41, 67], “lability and exacerbated emotional intensity” [24, 33, 34], but also “cyclothymia” [69, 70], and “borderline personality traits” [71]. Cyclothymic temperament is the most prevalent affective temperament in adults with ADHD [69, 70, 72, 73] and typical borderline symptoms are common in adults with ADHD [71]. However, it is unclear whether and how these aspects are related to ED in adults with ADHD. For example, some patients appear to have borderline personality traits [74], while others exhibit a cyclothymic temperament [69], suggesting that different patterns of ED could be observed. The aim of this study is to identify the clinical patterns of ED in adults with ADHD. To this end, a large cohort of newly diagnosed adults with ADHD completed self-reported questionnaires investigating several facets of ED symptoms. We also investigated ADHD symptoms more broadly, including clinical dimensions usually associated with ADHD (e.g., sleep difficulties and racing thoughts), and how ED features were associated with childhood maltreatment and functional impairment.

## Methods

### Participants

A total of 460 adults newly diagnosed with ADHD aged 18 to 76 (*M* = 33.89 years; *SD* = 10.76, 52.6% were women) were included in this cross-sectional study. Participants were recruited from the outpatient psychiatry clinics of the University Hospital of Strasbourg. Clinical assessments were led by senior psychiatrists and diagnoses were established according to the DSM-5 criteria for ADHD and comorbidities [4]. Inclusion criteria included being older than 18 years and the absence of a current acute depressive or manic phase. Demographic data, comorbidities and treatment were recorded at the diagnostic assessment. After the clinical interview, all patients completed several self-reported questionnaires assessing ADHD, other psychiatric disorders’ symptoms (e.g., borderline, depressive and anxiety symptoms) and additional clinical dimensions including ED questionnaires. We excluded subjects with missing data, thus the analyses were conducted on a sample of 391 adults with ADHD.

Subjects provided a written consent prior to inclusion in the study in accordance with the Declaration of Helsinki. This study was approved by the ethics committee of the Faculties of Medicine, Odontology, Pharmacy, Schools of Nursing, Physiotherapy, Maieutics and the University Hospitals of Strasbourg.

### Questionnaires

The Self-Reported Wender-Reimherr Adult Attention Deficit Disorder Scale (SR-WRAADDS; [75]) is a 61-item self-reported questionnaire assessing ADHD symptomatology, including inattention, hyperactivity, disorganization and emotional dysregulation (the 30 first items), and its impact on patients’ daily life (the 31 last items). A recent factor analysis conducted on the 30 items measuring ADHD-related symptoms in a sample of adults with ADHD yielded a four-factor model, encompassing: (i) attention/disorganization (items 1, 2, 3, 4, 5, 21, 22, 23, 24), (ii) hyperactivity/restlessness (items 7, 8, 9), (iii) impulsivity/emotional outbursts (items 10, 11, 12, 26, 29), (iv) emotional lability (items 13, 15, 17, 18, 19, 20) [26]. In this study, we only focus on the ADHD symptomatology and chose to use this new factor solution.

Impulsivity was assessed according to the five-factor model of impulsivity using the Urgency Premeditation Perseverance Sensation seeking-Positive urgency short version (UPPS-P; [76]). The UPPS-P is a 20-item scale comprising 4 items per impulsive dimension (i.e. “Urgency”, “Positive urgency”, “Lack of premeditation”, “Lack of perseverance”, “Sensation seeking”). Items are rated on a 4-point Likert scale ranging from 1 (“totally agree”) to 4 (“totally disagree”).

The Borderline Symptom List-23 (BSL-23; [77]) is a self-reported questionnaires assessing the severity of the typical borderline symptomatology. The BSL-23 comprises 23 items rated on a 5-point Likert scale ranging from 0 (“not at all”) to 4 (“very strong”). A total score is calculated including all the 23 items.

Depression symptoms were assessed by the Beck Depression Inventory (BDI; [78, 79]). The BDI is a 21-item self-reported questionnaire with items ranging from 0 (the symptom is absent) to 3 (maximum severity).

The brief Temperament Evaluation of Memphis, Pisa, Paris, and San Diego Autoquestionnaire (TEMPS-A; [80]) is the short version of the 110-item original questionnaire comprising 39 items, rated on a yes-or-no scale. The TEMPS-A is a self-reported questionnaire designed to assess affective temperaments via five subscales: (i) items 1 to 12 assess cyclothymic temperament, (ii) items 13 to 20 assess depressive temperament, (iii) items 21 to 28 assess irritable temperament, (iv) items 29 to 36 assess hyperthymic temperament, and (v) items 37 to 39 assess anxious temperament. To enhance response variability, the original TEMPS-A scale underwent adaptation. Each item was transformed into a 4-point Likert scale ranging from 0 ("not at all") to 3 ("a lot"), as opposed to the binary response format of a yes-or-no scale. This modification has broadened the range of responses.

The Racing and Crowded Thoughts Questionnaire-13-item (RCTQ-13; [81]) is a 13-item self-reported questionnaire assessing three facets of racing thoughts during the last 24 h – i.e., thought overactivation, burden of thought overactivation and thought overexcitability. Items are rated on a 5-point Likert scale ranging from 0 (“not at all”) to 4 (“totally agree”).

The Cognitive Processes Associated with Creativity (CPAC; [82]) is a 28-item self-reported questionnaire measuring the use of different cognitive strategies involved in the creative process. The CPAC encompasses 5 subscales i.e., idea manipulation, imagery, flow of ideas, metaphorical/analogical thinking, idea generation and idea incubation. Items are rated on a 5-point Likert scale ranging from 1 (“never”) to 5 (“always”).

The Insomnia Severity Index (ISI, [83]) is a 7-item self-reported questionnaire assessing insomnia symptoms, including type, severity and functional impact. Items are scored on a 5-point Likert scale ranging from 0 (“not at all”) to 4 (“extremely”).

The Epworth Sleepiness Scale (ESS; [84]) is a 8-item self-reported questionnaire assessing the propensity to be sleepy in 8 different daily situations (e.g. when watching TV, when reading a document, when having a conversation…). Items are scored on a 4-point Likert scale ranging from 0 (“weak probability”) to 3 (“high probability”).

The Childhood Trauma Questionnaire Short-form (CTQ-SF; [85]) was used to assess the different childhood maltreatment (e.g. sexual abuse, emotional neglect, emotional abuse, physical abuse and physical neglect) that might be involved in the severity of ADHD symptomatology. The CTQ-SF is a self-reported questionnaire comprising 28 items, rated on a 5-point Likert scale, ranging from 0 (“never”) to 4 (“very often”).

The Weiss Functional Impairment Rating Scale (WFIRS; [86, 87]), is a 69-item self-reported questionnaire used to assess functional impairment. The WFIRS assesses daily difficulties in ADHD in seven domains of functioning (family, work, school, life skills, self-concept, social functioning, and risky activities). Items are rated on a 4-point Likert scale, ranging from 0 (“never”) to 3 (“very often”).

### Statistical analyses

Descriptive statistics included frequencies and percentages of categorical variables together with means and standard deviations of continuous variables. To investigate the different possible symptoms clusters in our sample of adults with ADHD, a factor analysis with the principal component extraction method was performed [88]. Considering that the obtained factors were correlated, an oblique rotation (*Oblimin*) was applied. Factors were retained based on their eigenvalue (greater than 1) and after inspection of the scree plot. Only non-overlapping items with loadings greater than 0.40 were considered as being part of a factor. A mono-dimensional clustering was conducted for each principal component obtained to assess whether participants presented or not with each symptomatic factor [89]. This mono-dimensional clustering consisted in an unsupervised k-means clustering for which we predetermined 2 clusters. Following the analysis, a score of 0 was attributed to the cluster when the patient did not present with this specific symptoms whereas a score of 1 was attributed when the patient was concerned by the symptomatic cluster. The mono-dimensional clustering was based on each subject factor score for each of the 5 components calculated by the Jamovi® software.. Finally, two linear regression analyses were conducted. On the one hand, a multiple linear regression analysis was performed to investigate the contribution of the identified factors in functional impairment. On the other hand, a simple linear regression analysis was carried out to partial out the specific contribution of childhood maltreatment on the extracted symptomatic factors. Statistical significance was set at 0.05. Analyses were performed using the Jamovi® and MatLab® softwares.

## Results

### Demographic description

Among the 391 retained adults with ADHD, the combined presentation was the most prevalent, concerning 73.40% of the group, followed by the inattentive presentation and the hyperactive one (22.9% and 3.7% respectively). Regarding psychiatric comorbidities, 34.1% (*n* = 106) experienced a past episode of depression and 15.1% (*n* = 41) have a comorbid bipolar disorder. 14.8% (*n* = 16) adults of our sample had an anxiety disorder, 2.05% (*n* = 8) presented with an obsessive–compulsive disorder, 8% (*n* = 40) presented a borderline personality disorder and 4.5% (*n* = 14) an autism spectrum disorder. Concerning medications, 67% (*n* = 154) of patients did not take any psychotropic drugs at the time of the recruitment. Among those under medication at recruitment, 1.28% (*n* = 5) had just started their psychostimulant treatment, 18.4% (*n* = 52) were treated with antidepressants and 10.3% (*n* = 29) with anxiolytics. Moreover, 9.90% (*n* = 28) of the sample were taking mood stabilizers, 7.1% (*n* = 20) antipsychotics and 2.8% (*n* = 8) were under hypnotics. Detailed demographic data are presented in Table 1.

**Table 1:** Demographic and clinical characteristics of the total sample

**Variables**	**Total sample (*N* = 391)**
Age, Mean *(SD)*	33.57 *(10.90)*
Gender, *N (%)*	187 M *(47.8%)*
	203 F *(51.9%)*
	1 Transgender *(0.3%)*
Years of education, Mean *(SD)*	14.24 (*2.67)*
***ADHD presentation***	
Inattentive	75 *(22.9%)*
Hyperactive	12* (3.7%)*
Combined	240 *(73.4%)*
***Comorbidities****, N (%)*	
Past Major Depression Episode	106 *(27.11%)*
Bipolar Disorder	47* (12.02%)*
Anxiety Disorder	46* (11.76%)*
Obsessive Compulsive Disorder	8* (2.05%)*
Borderline Personality Disorder	25* (6.39%)*
Autism Spectrum Disorder	14* (3.58%)*
***Treatments****, N (%)*	
Psychostimulants	5 *(1.28%)*
Antidepressants	52* (13.30%)*
Anxiolytics	29* (7.42%)*
Mood Stabilizers	28* (7.16%)*
Antipsychotics	20* (5.12%)*
Hypnotics	8* (2.05%)*
***Questionnaires, M (SD)***	
CTQ-SF	21.62* (15.71)*
WFIRS – Family (*n* = *383)*	1.53* (0.779)*
WFIRS – Work (*n* = *318)*	1.532* (0.709)*
WFIRS – Studies (*n* = *178)*	1.766* (0.863)*
WFIRS – Life skills (*n* = *391)*	1.495* (0.570)*
WFIRS – Self-concept (*n* = *389)*	2.240* (0.843)*
WFIRS – Social functioning (*n* = *389)*	1.301* (0.677)*
WFIRS – Risky behaviors (*n* = *388)*	0.71* (0.53)*
***Symptomatic Clusters, N (%)***	
Emotional instability	218 *(55.75%)*
Impulsivity	197 *(50.38%)*
Overactivation	234* (59.85%)*
Inattention/Disorganization	204 *(52.17%)*
Sleep problems	199 *(50.90%)*

*Note: CTQ-SF* Childhood Trauma Questionnaire Short-Form, *WFIRS* Weiss Functional Impairment Rating Scale

### Factor analysis

A total of 20 variables were used to perform the factor analysis. The variables consisted in the four symptoms subscales of the SR-WRAADDS (i.e., attention/disorganization, hyperactivity/restlessness, impulsivity/emotional outbursts and emotional lability), the five UPPS-P subscales, the BSL-23, the BDI, the five TEMPS-A subscales, the RCTQ-13, the CPAC, the ISI and the ESS. The analysis yielded a five factors solution explaining 60.1% of the total variance. Each factor presented with an eigenvalue greater than 1 (eigenvalues 5.52, 2.09, 1.87, 1.39, 1.15 respectively), explaining 17.1%, 15.6%, 11.1%, 8.9% and 7.4% of the variance (Table 2). The first factor, labelled “*emotional instability*”, had strong factor loadings for the TEMPS-A-Depressive subscale, the BDI, the BSL-23, the SR-WRAADDS-Emotional lability subscale, the TEMPS-A-Cyclothymic subscale and the TEMPS-A-Anxious subscale. The second factor, “*impulsivity*” included the UPPS-P-Urgency subscale, the SR-WRAADDS-Impulsivity subscale, the UPPS-P-Positive urgency subscale, the UPPS-P-Lack of premeditation subscale and the TEMPS-A-Irritable subscale. The third factor, labelled “*overactivation*”, comprised the CPAC, the TEMPS-A-Hyperthymic subscale, the RCTQ-13 and the SR-WRAADDS-Hyperactivity subscale. The fourth factor, labelled “*inattention/disorganization*”, had strong factor loadings for the UPPS-P-Lack of perseverance subscale and the SR-WRAADDS-Attention/Disorganization subscale. The fifth factor, labelled “*sleep problems*”, consisted in the ISI and the ESE. The UPPS-P-Sensation seeking subscale was not considered because of its overlap between the “*impulsivity*” and the “*overactivation*” factors.

**Table 2:** Factor analysis

	FACTOR 1 Emotional instability	FACTOR 2 Impulsivity	FACTOR 3 Overactivation	FACTOR 4 Inattention/ disorganization	FACTOR 5 Sleep problems
*% Explained Variance*	*17.1%*	*15.6%*	*11.1%*	*8.9%*	*7.4%*
SR-WRAADDS-Attention/Disorganization				0.764	
SR-WRAADDS-Hyperactivity/Restlessness			0.417		
SR-WRAADDS-Impulsivity/Emotional outbursts		0.704			
SR-WRAADDS-Emotional lability	0.700				
UPPS-P-Urgency		0.788			
UPPS-P-Positive urgency		0.700			
UPPS-P-Lack of premeditation		0.688			
UPPS-P-Lack of perseverance				0.845	
UPPS-P-Sensations seeking		0.446	0.479		
BSL-23	0.664				
BDI	0.713				
TEMPS-A-Cyclothymic	0.532				
TEMPS-A-Depressive	0.830				
TEMPS-A-Irritable		0.592			
TEMPS-A-Hyperthymic			0.749		
TEMPS-A-Anxious	0.440				
RCTQ-13			0.553		
CPAC			0.809		
ISI					0.656
ESS					0.795

*Note: SR-WRAADDS* Self-Reported Wender-Reimherr Adult Attention Deficit Disorder Scale, *UPPS-P* Urgency Premeditation Perseverance Sensation seeking – Positive urgency Short version, *BSL-23* Borderline Symptoms List-23, *BDI* Beck Depression Inventory, *TEMPS-A* Temperament Evaluation of Memphis, Pisa, Paris, and San Diego Autoquestionnaire, *RCTQ-13* Racing and Crowded Thoughts Questionnaire-13 item, *CPAC* Cognitive Processes Associated with Creativity, *ISI* Insomnia Severity Scale, *ESS* Epworth Sleepiness Scale

The UPPS-P Sensation seeking subscale was excluded of the factor analysis because of its overlap between the “*impulsivity*” and the “*overactivation*” factors

### Mono-dimensional clustering

**Fig. 1:**
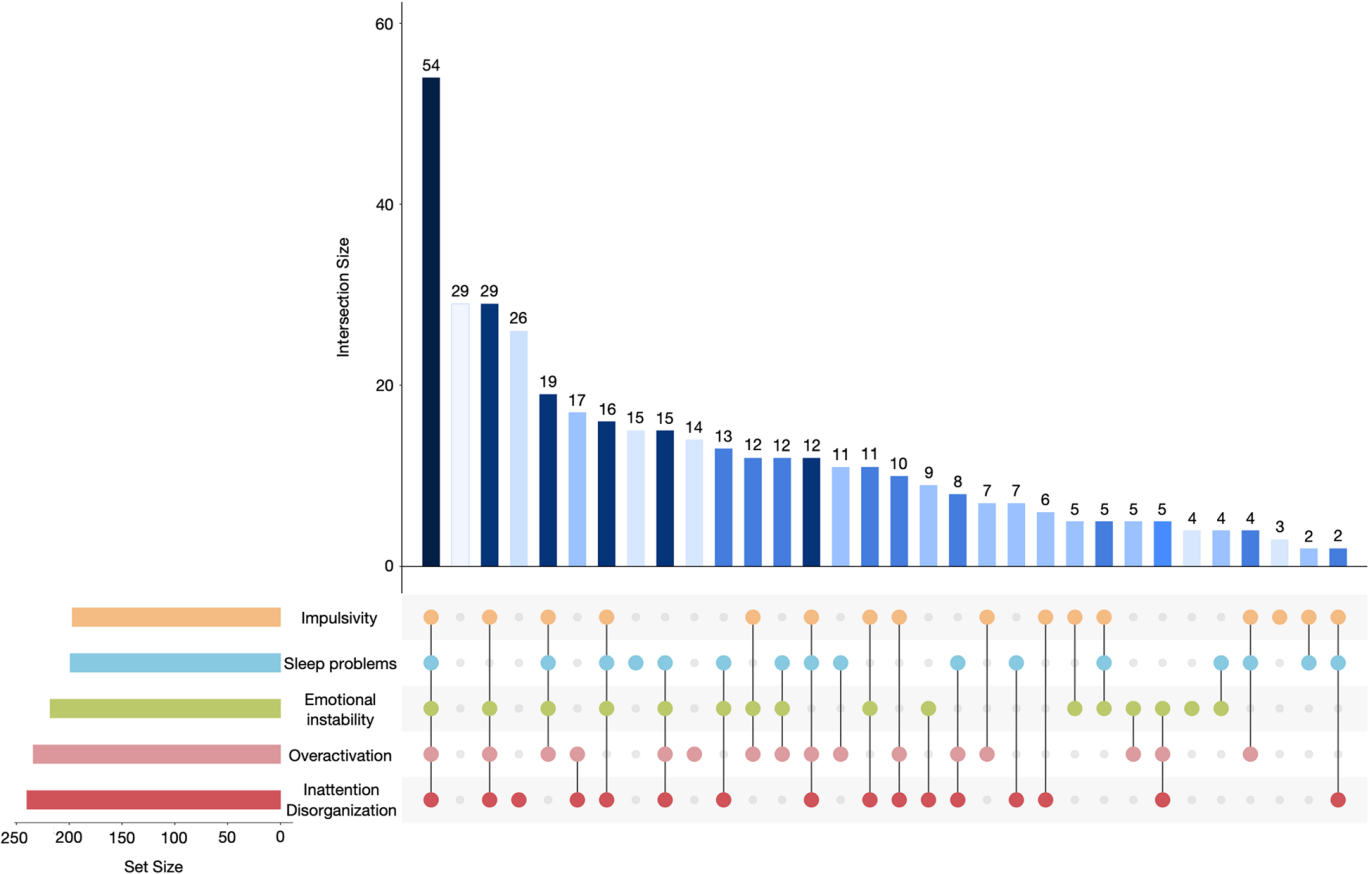
Upset plot representing the distribution of the symptomatic clusters in the total sample of 391 adults with ADHD

In total, 218 subjects presented the “*emotional instability*” cluster, 197 the “*impulsivity*” cluster, 234 the “*overactivation*” cluster, 240 the “*inattention/disorganization*” cluster and 199 the “*sleep problems*” cluster (Fig. 1). On the whole sample, only few patients presented with only one symptomatic cluster. 4, 3, 14, 26 and 15 participants respectively presented with just “*emotional instability*”, “*impulsivity*”, “*overactivation*”, “*inattention/disorganization*” or “*sleep problems*”. In most cases, adults with ADHD were attributed to several clusters (*n* = 300), whereas some patients do not present any symptomatic cluster (*n* = 29). The most prevalent combination was the one with the 5 factors (*n* = 54).

### Regression analyses

A multiple regression analysis was conducted to partial out the specific contribution of the five symptomatic clusters to the functional impairment of adults with ADHD. The five obtained factors were simultaneously entered as predictors in the model. Results are presented in Table 3. Regarding the 7 domains of life impairment of the WFIRS, predictors accounted for 8.9% to 37.2% of the variance. Regarding the WFIRS-Family subscale, the “*emotional instability*”, “*impulsivity*” and the “*sleep problems*” factors were the main predictor of scores (*β* = 0.37, *β* = 0.24 and *β* = 0.09; *p* < 0.05 respectively). These factors were also the main predictors for the WFIRS-Social functioning subscale (*β* = 0.34, *β* = 0.14 and *β* = 0.17; *p* < 0.05 respectively). The “*impulsivity*”, the “*inattention/disorganization*” and the “*sleep problems*” factors were the main predictors of the WFIRS-Work and WFIRS-Daily skills subscales (*β* = 0.28, *β* = 0.40, *β* = 0.13, and *β* = 0.34, *β* = 0.35, *β* = 0.18 respectively). Regarding the WFIRS-Self-concept subscale, the main predictors consisted in “*emotional instability*”, “*overactivation*” and the “*inattention/disorganization*” factors (*β* = 0.58, *β* = -0.11, and *β* = 0.19). For the WFIRS-Risky behaviors subscale the factors “*emotional instability*”, “*inattention/disorganization*” and “*overactivation*” were the main predictors (*β* = 0.09, *β* = 0.47, and *β* = 0.14).

**Table 3:** Multiple linear regression analysis on the contribution of each identified symptomatic cluster to the patients’ functional impairment

	WFIRS –Family^a^ (*n* = 383)		WFIRS –Work^b^ (*n* = 318)		WFIRS – Studies^c^ (*n* = 178)		WFIRS –Life skills^d^ (*n* = 391)		WFIRS –Self-concept^e^ (*n* = 389)		WFIRS – Social functioning^f^ (*n* = 389)		WFIRS –Risky Behaviors^g^ (*n* = 388)	
	*β*	*p*	*β*	*p*	*β*	*p*	*β*	*p*	*β*	*p*	*β*	*p*	*β*	*p*
Factor 1 – Emotional instability	0.367	** < .001**	0.279	** < .001**	0.044	.56	0.338	** < .001**	0.579	** < .001**	0.344	** < .001**	0.090	**.05**
Factor 2 – Impulsivity	0.235	** < .001**	-0.077	.15	0.063	.42	0.056	0.21	-0.026	.56	0.143	**.004**	0.467	** < .001**
Factor 3 – Overactivation	0.056	.21	0.036	.47	0.089	.23	0.05	0.24	-0.114	**.006**	-0.035	.45	0.144	**.001**
Factor 4 – Inattention/disorganization	0.042	.35	0.401	** < .001**	0.292	** < .001**	0.348	** < .001**	0.192	** < .001**	-0.013	.78	-0.011	.80
Factor 5 – Sleep problems	0.096	**.03**	0.134	**.007**	0.033	.65	0.178	** < .001**	0.002	.96	0.174	** < .001**	0.036	.41

*p*< .05; *WFIRS* Weiss Functional Impairment Rating Scale

^a^*R*^*2*^ = 0.285; *adjusted R*^*2*^ = 0.275; *F*(377,5) = 30.03; *p* < .001

^b^*R*^*2*^ = 0.282; *adjusted R*^*2*^ = 0.270; *F*(312,5) = 24.47; *p* < .001

^c^*R*^*2*^ = 0.115; *adjusted R*^*2*^ = 0.089; *F*(172,5) = 4.46; *p* < .001

^d^*R*^*2*^ = 0.346; *adjusted R*^*2*^ = 0.337; *F*(385,5) = 40.72; *p* < .001

^e^*R*^*2*^ = 0.380; *adjusted R*^*2*^ = 0.372; *F*(383,5) = 47.04; *p* < .001

^f^*R*^*2*^ = 0.216; *adjusted R*^*2*^ = 0.206; *F*(383,5) = 21.11; *p* < .001

^g^*R*^*2*^ = 0.305; *adjusted R*^*2*^ = 0.296; F(382,5) = 33.58; *p* < .001

To partial out the specific contribution of childhood maltreatment to the obtained factors (i.e. emotional lability, impulsivity, overactivation, inattention/disorganization and sleep problems) a simple regression was conducted using the CTQ-SF as a predictor variable. Results are presented in Table 4. Regarding the “*emotional instability*”, the ‘*impulsivity*” and the “*sleep problems*” factors, the CTQ-SF accounted respectively for 10.6%, 4.5% and 2.4% of the variance (*F* = 47.08; *p* < 0.001; *F* = 19.46, *p* < 0.001; *F* = 10.41, *p* = 0.001).

**Table 4:** Simple linear regression analysis highlighting the contribution of childhood maltreatment to the five clusters of ADHD symptomatology

	*Factor 1* Emotional Instability^a^		*Factor 2* Impulsivity^b^		*Factor 3* Overactivation^c^		*Factor 4* Inattention/Disorganization^d^		*Factor 5* Sleep problems^e^	
	*β*	*p*	*β*	*p*	*β*	*p*	*β*	*p*	*β*	*p*
CTQ-SF	0.329	** < .001**	0.219	** < .001**	0.064	.21	0.017	.74	0.162	**.001**

*CTQ-SF* Childhood Trauma Questionnaire Short-Form

^a^*R*^*2*^ = 0.108; *adjusted R*^*2*^ = 0.106; *F*(388,1) = 47.081; *p* < .001

^b^*R*^*2*^ = 0.048; *adjusted R*^*2*^ = 0.045; *F*(388,1) = 19.458; *p* < .001

^c^*R*^*2*^ = 0.064; *adjusted R*^*2*^ = 0.004; *F*(388,1) = 1.575; *p* = .210

^d^*R*^*2*^ = 0.017; *adjusted R*^*2*^ = -0.002; *F*(388,1) = 0.112; *p* = .738

^e^*R*^*2*^ = 0.026; *adjusted R*^*2*^ = 0.024; *F*(388,1) = 10.410; *p* = .001

## Discussion

This study aimed at identifying the clinical patterns of ED in adults with ADHD, through the use of self-reported questionnaires assessing several psychological dimensions potentially related to ED. Overall, our results suggest that the clinical presentation of adults with ADHD is broader than the classic symptomatic triad comprising inattention and hyperactivity-impulsivity [4]. Indeed, using factor analysis, the sets of symptoms found here are highly similar to the clinical picture of adult ADHD described by Asherson [12], and include ED, overactivity, and sleep problems. Indeed, the factor analysis revealed five symptomatic dimensions which seem to account for the clinical presentation of most adult ADHD cases. Emotional difficulties were part of three out of five clusters, suggesting that this symptom is central in the clinical picture of adult ADHD [14, 31, 34, 42, 63, 90]. The first cluster, i.e., “*emotional instability*”, refers to emotional fluctuations and the vulnerability to negative emotions. The second cluster, i.e., “*impulsivity*”, refers to the excessive emotional reactions in response to both positive and negative emotional situations and is in line with the concept of emotional impulsivity described by Barkley and Fischer [40] and Barkley [91]. The third cluster, labelled “*overactivation*”, encompasses several strengths of functioning of adults with ADHD, including hyperthymic temperament traits, racing thoughts and creativity. This factor is akin to the positive emotionality factor recently highlighted in the literature [92]. The other two factors were independent of emotional difficulties. The fourth and the fifth clusters, respectively labelled “*inattention/disorganization*” and “*sleep problems*”, refer to attention and cognitive problems and sleep difficulties.

Our results are in line with those of prior studies using factor analysis which suggest that inattention, associated with executive dysfunction, is a robust feature of ADHD [26, 35, 67, 75, 92, 93]. Indeed the factor “*inattention/disorganization*” concerns 61.38% of our sample of adults with ADHD. This number is similar to that referring to the “*emotional instability*” and/or “*impulsivity*” factors, as 67.52% of our sample presented with at least one of these symptoms. Interestingly, some authors suggest that considering two ADHD phenotypes (i.e. an inattention presentation and a combined inattention and dysregulated emotions presentation) may be more accurate than the classic three phenotypes (inattention, hyperactive-impulsive and combined presentations) [35, 75]. Regarding studies supporting the centrality of ED in adult ADHD [14, 26, 31, 34, 35, 42, 63, 90, 94], our results add to those and are the first using several measures of ED. Moreover, consistent with a number of recent studies on ED in ADHD, our results indicate that a large majority of individuals identified as presenting the two ED factors had the combined presentation (82.03%) compared to the inattentive presentation (14.84%; 24,35,67).

Regarding the patterns of ED found in adult ADHD, consistent with the study led by Weibel et al. [26], we found that ED can be conceptualized along two dimensions: the emotional instability dimension, associated with vulnerability to sadness, sensitivity to environmental stressors, and their behavioral implications, and the emotional impulsivity dimension. However, those two dimensions rarely occur alone. Indeed, only 17.14% and 11,76% of the participants were concerned with the “*emotional instability*” or the “*impulsivity*” factor alone. This can be explained by the temporal model of emotions symptoms in ADHD [63]. In this model, excessive experience or high sensitivity to emotional stimulations and further regulation strategies are distinguished. Faraone et al. [63] posit that emotional instability and emotional impulsivity are inherently associated, resulting from a hypersensitive emotional generation, whereas deficient late emotion regulation strategies are rather involved in the slower return to an emotional neutral state. Of note, emotional impulsivity and emotional lability are thought to be the main features of the ED experience in the ADHD combined presentation [63]. In contrast, deficient emotional self-regulation without emotional impulsivity is hypothesized to be more specifically associated with the ADHD inattentive presentation [63]. In line with Faraone et al.’s assumptions [63], we found that the combined ADHD presentation was the most prevalent among patients presenting with both “*emotional instability*” and “*impulsivity*” factors (82.17%), while the inattentive ADHD presentation concerned only 14.73% of those patients. This suggests that different mechanisms may underpin the experience of ED in the combined and the inattentive presentation of ADHD. Furthermore, consistent with a number of studies, the ED factors contributed to most functional difficulties of adults with ADHD, i.e. behavioral, academic and social impairment [39, 40, 92, 95, 96]. For instance, in a recent factor analysis study, Pallucchini et al., [92] also identify an ED factor, and patients with ED presented with the most severe impairments. In addition to the contribution of ED to the functional impairment associated with adult ADHD, our results suggest a weak, albeit significant contribution of childhood maltreatment to ED and sleep problems. This is consistent with a number of studies suggesting that environmental factors and especially childhood maltreatment are involved in the development and the severity of ED in ADHD [54], similar to other disorders, such as BPD and BD [54, 97, 98].

In contrast to ED, the factor “*overactivation*” was positively associated with increased self-esteem and did not contribute to any functional difficulties. Importantly, there is a growing number of studies focusing on the strengths of the ADHD functioning, shifting therefore from the deficit-focus view of ADHD [99–101]. Creativity, racing thoughts, motor hyperactivity and hyperthymic temperament are the dimensions composing the “*overactivation*” factor. Interestingly, in a qualitative study on the ADHD strengths, Sedgwick et al. [101] found that racing thoughts and creativity belonged to a global theme, labelled cognitive dynamism. Cognitive dynamism included ceaseless mental activity akin to racing thoughts found in BD [36, 101, 102]. Furthermore, Segdwick et al. [101] also found that motor hyperactivity may be beneficial. Indeed, increased physical energy leads patients to engage in physical activities, which may, in turn, contribute to their well-being. In line with the positive emotionality factor identified by Pallucchini et al. [92], consisting of few emotional control difficulties, the hyperthymic temperament was part of our “*overactivation*” factor. Individuals presenting with hyperthymic temperament display exuberance, tirelessness, sensation seeking and a high level of energy [80]. Although the hyperthymic temperament is not among the most prevalent affective temperament in adults with ADHD [70, 72], it could characterize a subgroup of high-functioning people with ADHD whom present with limited functional impairments [99]. Indeed, in the general population, the hyperthymic temperament has been found to have a protective effect against the development of mental health issues [103], such as addiction [104, 105]. Moreover, in adults with bipolar disorder, hyperthymic temperament traits are a protective factor against relapse, severity of anxiety and suicidality [106]. Overall, individuals presenting with the “*overactivation*” factor, reported more positive aspects of ADHD and fewer emotional difficulties, which are involved in the functional impairment associated with ADHD.

We acknowledge some limitations to this study. First, given the naturalistic approach used, the adults with ADHD recruited presented with different comorbidities, which may have influenced the factor analysis. Secondly, since our sample of adults with ADHD was recruited in a clinical setting, it included people who sought care and were likely to present with significant functional impairment,. Hence, participants probably experienced more severe ADHD and ED symptoms than the general adult ADHD population, limiting the generalization of our results to the general ADHD. For instance, the overactivation features might be more prominent in the general ADHD community, including adults who have a satisfactory quality of life. A replication of this study in the general population is warranted to capture a broader spectrum of ADHD and ED symptoms severity and to confirm the distribution of symptomatic factors more generally. Third, given the sample size, we were unable to perform a factor analysis on the items of each questionnaire and used the global scale or subscales scores. An item-based analysis could yield a more accurate description of ADHD. Nevertheless, this study counted a total of 20 measures allowing a large scanning of the ADHD functioning. Future studies should investigate the relative importance of each symptom using a network analysis approach. As an example Silk et al. [107] used this statistical method on the 18 ADHD diagnostic criteria described in the DSM-5 and found that motor hyperactivity is as central as the tendency of losing objects in ADHD.

## Conclusions

In this study, we identified five symptomatic clusters that characterized adults with ADHD. Among them, ED appears as a central bi-factorial symptom, encompassing emotional lability and emotional impulsivity, contributing to greater functional impairment and partially explained by childhood maltreatment. However, adult ADHD cannot be reduced to a collection of clinical impairing symptoms, given that several features can be characteristic of high functioning ADHD and be involved in compensatory strategies used to overcome difficulties. In sum, there are relevant features beyond the classical symptomatic triad that may have significant implications for ADHD identification in adults. However, beyond functional impairment, ADHD may also be characterized by functional strengths.

## Data Availability

The datasets used and/or analyzed during the current study are available from the corresponding author on reasonable request.

## Abbreviations

ADHD: Attention Deficit Hyperactivity Disorder
BDI: Beck Depression Inventory
BSL-23: Borderline Symptom List-23
CPAC: Cognitive Processes Associated with Creativity
CTQ-SF: Childhood Trauma Questionnaire Short-Form
ED: Emotion Dysregulation
ESS: Epworth Sleepiness Scale
ISI: Insomnia Severity Scale
RCTQ-13: Racing and Crowded Thoughts Questionnaire-13 item
SR-WRAADDS: Self-Reported Wender-Reimherr Adult Attention Deficit Disorder Scale
TEMPS-A: Temperament Evaluation of Memphis, Pisa, Paris, and San Diego Autoquestionnaire
UPPS-P: Urgency Premeditation Perseverance Sensation seeking Positive urgency
WFIRS: Weiss Functional Impairment Rating Scale
